# *Streptococcus* Species Abundance in the Gut Is Linked to Subclinical Coronary Atherosclerosis in 8973 Participants From the SCAPIS Cohort

**DOI:** 10.1161/CIRCULATIONAHA.123.063914

**Published:** 2023-07-12

**Authors:** Sergi Sayols-Baixeras, Koen F. Dekkers, Gabriel Baldanzi, Daniel Jönsson, Ulf Hammar, Yi-Ting Lin, Shafqat Ahmad, Diem Nguyen, Georgios Varotsis, Sara Pita, Nynne Nielsen, Aron C. Eklund, Jacob B. Holm, H. Bjørn Nielsen, Ulrika Ericson, Louise Brunkwall, Filip Ottosson, Anna Larsson, Dan Ericson, Björn Klinge, Peter M. Nilsson, Andrei Malinovschi, Lars Lind, Göran Bergström, Johan Sundström, Johan Ärnlöv, Gunnar Engström, J. Gustav Smith, Marju Orho-Melander, Tove Fall

**Affiliations:** Molecular Epidemiology and Science for Life Laboratory (S.S.-B., K.F.D., G. Baldanzi, U.H., Y.-T.L., S.A., D.N., G.V., T.F.), Department of Medical Sciences, Uppsala University, Sweden.; Clinical Physiology (A.M.), Department of Medical Sciences, Uppsala University, Sweden.; Clinical Epidemiology (L.L., J.S.), Department of Medical Sciences, Uppsala University, Sweden.; CIBER Cardiovascular Diseases (CIBERCV), Instituto de Salud Carlos III, Madrid, Spain (S.S.-B.).; Department of Clinical Sciences in Malmö, Lund University, Sweden (D.J., U.E., L.B., F.O., A.L., P.M.N., G.E., M.O.-M.).; Public Dental Service of Skåne, Lund, Sweden (D.J.).; Departments of Periodontology (D.J., B.K.), Faculty of Odontology, Malmö University, Sweden.; Cariology (D.E.), Faculty of Odontology, Malmö University, Sweden.; Division of Family Medicine and Primary Care, Department of Neurobiology, Care Science and Society, Karolinska Institutet, Huddinge, Sweden (Y.-T.L., J.Ä.).; Preventive Medicine Division, Harvard Medical School, Brigham and Women’s Hospital, Boston, MA (S.A.).; Clinical Microbiomics A/S, Copenhagen, Denmark (S.P., N.N., A.C.E., J.B.H., H.B.N.).; The Novo Nordisk Foundation Center for Biosustainability, Technical University of Denmark, Lyngby, Denmark (S.P.).; Clinical Studies Sweden, Forum Söder, Region Skåne, Lund, Sweden (L.B.).; Section for Clinical Mass Spectrometry, Danish Center for Neonatal Screening, Department of Congenital Disorders, Statens Serum Institut, Copenhagen, Denmark (F.O.).; Department of Dental Medicine, Karolinska Institutet, Solna, Sweden (B.K.).; Department of Internal Medicine, Skåne University Hospital, Malmö, Sweden (P.M.N.).; Department of Molecular and Clinical Medicine, Institute of Medicine, Sahlgrenska Academy, University of Gothenburg, Sweden (G. Bergström).; Department of Clinical Physiology, Sahlgrenska University Hospital, Region Västra Götaland, Gothenburg, Sweden (G. Bergström).; The George Institute for Global Health, University of New South Wales, Sydney, Australia (J.S.).; School of Health and Social Studies, Dalarna University, Falun, Sweden (J.Ä.).; The Wallenberg Laboratory/Department of Molecular and Clinical Medicine, Institute of Medicine, Gothenburg University, Sweden (J.G.S.).; Department of Cardiology, Sahlgrenska University Hospital, Gothenburg, Sweden (J.G.S.).; Department of Cardiology, Clinical Sciences, and Wallenberg Center for Molecular Medicine and Lund University Diabetes Center, Lund University, Sweden (J.G.S.).; Skåne University Hospital, Lund, Sweden (J.G.S.).

**Keywords:** atherosclerosis, gastrointestinal microbiome, metagenomics, *Streptococcus*, tomography

## Abstract

Gut microbiota have been implicated in atherosclerotic disease, but their relation with subclinical coronary atherosclerosis is unclear. This study aimed to identify associations between the gut microbiome and computed tomography–based measures of coronary atherosclerosis and to explore relevant clinical correlates.

**METHODS:**

We conducted a cross-sectional study of 8973 participants (50 to 65 years of age) without overt atherosclerotic disease from the population-based SCAPIS (Swedish Cardiopulmonary Bioimage Study). Coronary atherosclerosis was measured using coronary artery calcium score and coronary computed tomography angiography. Gut microbiota species abundance and functional potential were assessed with shotgun metagenomics sequencing of fecal samples, and associations with coronary atherosclerosis were evaluated with multivariable regression models adjusted for cardiovascular risk factors. Associated species were evaluated for association with inflammatory markers, metabolites, and corresponding species in saliva.

**RESULTS:**

The mean age of the study sample was 57.4 years, and 53.7% were female. Coronary artery calcification was detected in 40.3%, and 5.4% had at least 1 stenosis with >50% occlusion. Sixty-four species were associated with coronary artery calcium score independent of cardiovascular risk factors, with the strongest associations observed for *Streptococcus anginosus* and *Streptococcus oralis* subsp *oralis* (*P*<1×10^–5^). Associations were largely similar across coronary computed tomography angiography–based measurements. Out of the 64 species, 19 species, including streptococci and other species commonly found in the oral cavity, were associated with high-sensitivity C-reactive protein plasma concentrations, and 16 with neutrophil counts. Gut microbial species that are commonly found in the oral cavity were negatively associated with plasma indole propionate and positively associated with plasma secondary bile acids and imidazole propionate. Five species, including 3 streptococci, correlated with the same species in saliva and were associated with worse dental health in the Malmö Offspring Dental Study. Microbial functional potential of dissimilatory nitrate reduction, anaerobic fatty acid β-oxidation, and amino acid degradation were associated with coronary artery calcium score.

**CONCLUSIONS:**

This study provides evidence of an association of a gut microbiota composition characterized by increased abundance of *Streptococcus* spp and other species commonly found in the oral cavity with coronary atherosclerosis and systemic inflammation markers. Further longitudinal and experimental studies are warranted to explore the potential implications of a bacterial component in atherogenesis.

**Clinical Perspective**

**What Is New?**

- Shotgun metagenomics identified associations between gut species and subclinical atherosclerosis assessed with computed tomography–derived coronary artery calcium score in 8973 participants, with an overrepresentation of the *Streptococcus* and *Oscillobacter* genera.
- Gut *Streptococcus* spp were positively associated with circulating biomarkers of systemic inflammation and infection response, and with the same species located in the mouth, which were in turn associated with oral pathologies.
- A subset of the coronary artery calcium score–associated species were negatively associated with indole propionate, but positively associated with secondary bile acids and imidazole propionate.

**What Are the Clinical Implications?**

- We describe the link between gut microbiota composition, especially species commonly found in the mouth, and subclinical coronary atherosclerosis and biomarkers of inflammation in the largest cardiovascular and metagenomics study to date.
- The effects of gut and oral *Streptococcus* spp on risk for coronary artery disease merit further longitudinal and experimental studies.

Atherosclerotic cardiovascular disease (CVD) is a major cause of death and disability.^1^ The microbial community of the gastrointestinal tract, also known as the gut microbiota, is hypothesized to affect the progression of atherosclerosis through 3 potential mechanisms. First, microbial metabolites can interfere with the host metabolism, including lipid metabolism.^2^ The composition of the gut microbiota has been linked to metabolic disorders such as obesity, insulin resistance, and type 2 diabetes, although the causal relation and direction are unclear.^3^ Second, translocation of live bacteria or bacterial structural components (eg, endotoxins) into the bloodstream can contribute to low-grade systemic inflammation, thereby exacerbating the atherosclerosis process.^2^ Third, the discovery of bacterial DNA within atherosclerotic plaques has led to the proposal that bacteria might directly infect plaques and accelerate atherosclerosis progression.^4^ Case–control studies of symptomatic coronary atherosclerotic disease have identified differences in the abundance of >500 gut species.^5–7^ However, dissimilarities in medical treatment and lifestyle factors of patients and controls make these studies prone to bias. Thus, studies of individuals without overt coronary disease in large population-based samples incorporating biomarkers of metabolism and inflammation are warranted.

Recent evidence supports that oral species are commonly transmitted to the gut,^8^ indicating that the gut and oral microbiota are connected rather than being 2 separate microbial communities. Causative species of dental caries, plaque-dwelling bacteria, and endocarditis-associated species including species from the viridans group streptococci (VGS) are reported to be transmitted at a high rate. In addition, an overlap is reported between oral bacteria such as *Veillonella* spp and *Streptococcus* spp found in the oral cavity, fecal samples, and carotid atherosclerotic plaque.^9^ Given the association between dental health and endothelial dysfunction and atherosclerotic disease,^10–12^ the gut may serve as a niche or entry route for oral pathogenic bacteria passing to the blood. However, there are few studies assessing the association of atherosclerosis-related gut species with the oral microbiome.

To avoid the limitations of previous studies and gain insight into the relation of the gut microbiota and atherosclerosis, we aimed to identify associations between the gut microbiome and computed tomography–based measures of subclinical coronary atherosclerosis in a large cohort of middle-aged participants from SCAPIS (Swedish Cardiopulmonary Bioimage Study). We further assessed associations between atherosclerosis-associated gut bacterial species and biomarkers of inflammation and infection, plasma metabolites, and the abundance of corresponding bacterial species in the oral cavity.

## METHODS

### Data Availability

Deidentified metagenomic sequencing data for SCAPIS samples can be accessed from the European Nucleotide Archive under accession number PRJEB51353 (https://www.ebi.ac.uk/ena/browser/view/prjeb51353). Access to pseudonymized SCAPIS phenotype data requires ethical approval from the Swedish Ethical Review Board and approval from the SCAPIS Data access board (https://www.scapis.org/data-access). Access to pseudonymized MOS (Malmö Offspring Study)/MODS (Malmö Offspring Dental Study) microbiome and phenotype data requires ethical approval from the Swedish Ethical Review Board and approval from the data access board (https://www.malmo-kohorter.lu.se/uttag). The source code and the summary data underlying all figures used to generate the results for the analysis are available at https://github.com/MolEpicUU/GUTSY_CACS.

### Study Design and Participants

**Figure 1.**
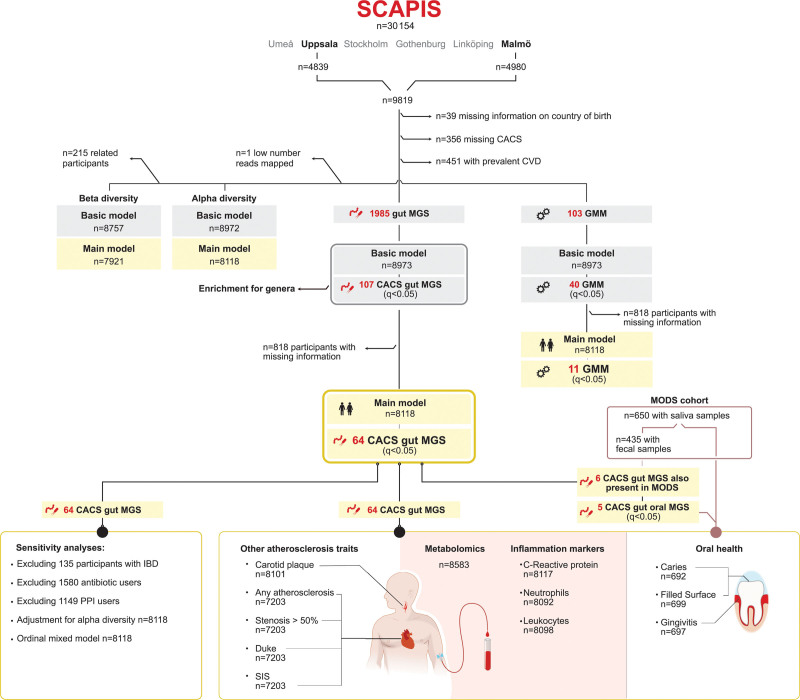
**Flowchart of the overall study design.** CACS indicates coronary artery calcium score; CVD, cardiovascular disease; GMM, gut metabolic module; IBD, inflammatory bowel disease; MGS, metagenomics species; MODS, Malmö Offspring Dental Study; PPI, proton-pump inhibitor; SCAPIS, Swedish Cardiopulmonary Bioimage Study; and SIS, segment involvement score.

The study design is illustrated in Figure 1. The primary data source was SCAPIS, a population-based study focusing on cardiovascular and respiratory disease in 30 154 participants invited between 50 and 64 years of age from 6 sites in Sweden.^13^ Fecal metagenomics data were available for 4839 participants from Uppsala and 4980 from Malmö after removal of 10 samples for failing quality control. After excluding participants with missing information on country of birth (n=39) or coronary artery calcium score (CACS; n=356), or who had self-reported CVD (n=451), including atherosclerotic CVD (n=119), 4541 participants from Uppsala and 4432 from Malmö remained.

The association between gut species and their counterparts in saliva samples and with oral health was investigated in participants of MODS (n=831; mean age, 52.9 years), a substudy of the family-based MOS.^10,14^ Salivary metagenomics, oral examination data, and complete data on age and sex were available for 650 participants; informative fecal metagenomics data from MOS were available for 435 of those participants.

All participants gave written informed consent. The study was conducted in accordance with the Declaration of Helsinki and was approved by the regional ethics committees (SCAPIS: Dnr 2010-228-31M and Dnr 2018/315; MODS: Dnr 2013/761; MOS: Dnr 2012/594).

### Atherosclerosis Measurements

Cardiac imaging in SCAPIS included coronary computed tomography angiography, as described previously.^15^ In brief, computed tomography was performed with a dedicated dual-source scanner and a Stellar Detector (Somatom Definition Flash; Siemens Medical Solutions). Noncontrast images for calcium scoring were obtained from all participants, followed by contrast-enhanced images from participants without contraindications. CACS was determined using syngo.via calcium scoring software (Volume Wizard; Siemens). The area of calcification was summed for the whole coronary artery tree to a CACS according to Agatston.^16^ Radiologists inspected the images for any coronary atherosclerosis, segment involvement score (SIS), Duke prognostic coronary artery disease index modified for SCAPIS,^15^ and occlusive atherosclerosis (≥50% stenosis in at least 1 vessel; Expanded Methods in the Supplemental Material). We included individuals with valid readings in at least 4 proximal segments. Carotid atherosclerosis was categorized as absent, unilateral, or bilateral^13^ from 2‐dimensional grayscale ultrasound images obtained with an Acuson S2000 ultrasound scanner and a 9L4 linear transducer (both from Siemens) using a standardized protocol and the Mannheim consensus.^17^

### Metagenomics

DNA was extracted from fecal samples (SCAPIS and MOS) and saliva samples (MODS) at Clinical Microbiomics A/S. The analytical pipeline is described in detail in the Expanded Methods in the Supplemental Material. In brief, libraries of fragmented DNA were sequenced with the Illumina NovaSeq 6000 platform. This technique generated an average of 26.3 million read pairs per sample in SCAPIS-Malmö and MOS, 25.3 million read pairs in SCAPIS-Uppsala, and 26.3 million read pairs in MODS. Non-host reads and data from these and other cohorts were used to build separate nonredundant gene catalogues for fecal and saliva samples. Metagenomic species and corresponding signature gene sets were identified.^18^ In all 3 data sets, species abundance was estimated by mapping reads to the signature gene sets and normalization for effective gene length. The taxonomic information for the 2 catalogues was mapped with the National Center for Biotechnology Information RefSeq database.^19^

Alpha diversity (Shannon diversity index, inverse Simpson index, and Chao1) and beta diversity (Bray-Curtis dissimilarity) were estimated with the R version 4.1.1 package vegan version 2.5-7 using rarefied data. Rarefied data were also used to define individuals with more than or less than median abundance of each species. The functional potential profile of the gut microbiota was determined by assigning genes to gut metabolic modules (GMM),^20^ which includes 103 metabolic pathways that represent cellular enzymatic processes. GMM abundance was estimated using the Omixer-RPM version 0.3.226 R package, with a minimum module coverage threshold set at 66.6%. Before statistical analysis, the abundance data for each species in all analytical data sets and for each GMM were transformed with the formula ln(*x*+1), where ln indicates natural logarithm and *x* denotes the relative abundance of each species or GMM, followed by *z* transformation to set the mean to 0 and SD to 1.

### Clinical Chemistry, Hematology, and Metabolomics

Venous plasma samples were analyzed for high-density lipoprotein and low-density lipoprotein cholesterol level, triglycerides, and high-sensitivity C-reactive protein (hsCRP). Blood cells were counted by standard methods. CACS-associated species–plasma metabolite associations in 8583 participants from SCAPIS with *q*<0.05 were accessed from the GUTSY atlas (https://gutsyatlas.serve.scilifelab.se).^21^ In brief, venous blood samples were collected from the participants after an overnight fast and stored at −80°C. The samples were then sent to Metabolon for plasma metabolome profiling. Proteins were removed from the samples and 4 different processes were used to analyze the metabolites: reverse phase/ultrahigh performance liquid chromatography–tandem mass spectroscopy with negative-ion mode electrospray ionization, hydrophilic interaction chromatography/ultrahigh performance liquid chromatography–tandem mass spectroscopy, and 2 separate resolutions with positive-ion mode electrospray ionization. Peak identification and quantification, as well as quality control, were performed using Metabolon hardware and software. Metabolites were annotated by matching them to a library of standards. A total of 1321 metabolites passed quality control and were included in the analyses. Association of microbial species with metabolites was assessed with partial Spearman rank correlation analysis adjusted for age, sex, place of birth (Scandinavia, non-Scandinavian Europe, Asia, or other), and technical variables.

### Oral Health Phenotypes

Dental examinations of MODS participants were done by 5 dentists. Surfaces with caries and fillings were recorded on all teeth, counting 4 to 5 surfaces per tooth. Surfaces with caries were detected using standard clinical criteria aided by mirror, probe (Hu-Friedy EXD57), and bitewing radiographs combining initial and manifest lesions. Filled surfaces included both fillings and crowns. Gingival inflammation was recorded as percentage of bleeding on probing, excluding wisdom teeth, and counting 6 surfaces per tooth.

### Other Phenotypes

In SCAPIS and MOS, data on country of birth, smoking, physical activity, and diet were collected with validated standardized questionnaires. Blood pressure and body mass index were recorded. Data on medications were acquired from the drug prescription register, self-report, plasma measurements, and other sources. Relatedness in SCAPIS was on the basis of kinship analysis of genotype data, assigning first-degree relatives to the same family, and relatedness in MOS/MODS was on the basis of family information from the study design phase. Collected phenotypes are described in detail in the Expanded Methods in the Supplemental Material.

### Statistical Analysis

#### Association Between Gut Microbiota Diversity and Atherosclerosis

The association of alpha diversity with coronary atherosclerosis was assessed by linear mixed models with ln(CACS+1) as the dependent variable and alpha diversity indices as the independent variables. To address the potential influence of family relatedness, the first-degree–relatedness among 423 participants from 208 families was accounted for with a random-effect variable by using the lmerTest version 3.1-3 R package. Furthermore, 2 sets of covariates were modeled as fixed effects. The basic model was adjusted for age, sex, country of birth, and metagenomics extraction plate. The main model was further adjusted for smoking; physical activity; energy-adjusted carbohydrate, protein, and fiber intake; systolic and diastolic blood pressure; total cholesterol level; high-density lipoprotein and low-density lipoprotein cholesterol levels; ln(triglycerides); diabetes; body mass index; and self-reported medication for dyslipidemia, hypertension, or diabetes. Correlation of covariates and alpha diversity measurements are reported in Figure S1.

Beta diversity was assessed by a distance-based multivariate ANOVA of ln(CACS+1) as the independent variable using the dmanova function in the GUniFrac version 1.3 package. These analyses were adjusted for the same 2 sets of covariates as used in the alpha diversity analyses. However, in contrast to the alpha diversity analyses, only 1 participant from each family cluster was included, rather than by treating family relatedness as a random-effect variable.

#### Association of Species and Bacterial Functions With CACS

Extensive simulations were conducted before the analysis and supported the use of linear regression to minimize false-positive findings (Expanded Methods in the Supplemental Material and Figure S2). Hence, a series of linear mixed multivariable regressions were performed with ln(CACS+1) as the dependent variable and the abundance of each species and GMM as the independent variable. The models were fitted for each species and each GMM separately; covariates from the basic model were used as fixed effects and family relatedness as random effects. Multiple testing was accounted for using the Benjamini-Hochberg false discovery rate at 5%.^22^ A taxon set enrichment analysis^23^ using the fgsea version 1.18.0 R package on the basis of *P* value ranking separately for positive and negative regression coefficients was used to determine whether certain genera were enriched.

Species with *q*<0.05 in the basic model were assessed adjusting for main model covariates, again accounting for multiple testing. To determine whether associations were caused by influential observations, unscaled dfbeta values were calculated using the influence and dfbetas R functions. The linear regression estimates were deemed unreliable if the exclusion of the observations with highest absolute dfbeta value resulted in a change of the direction of the regression coefficient or in *P*≥0.05. Findings not caused by an influential observation are referred to as CACS-associated species. Effect estimate modification by sex was assessed for CACS-associated species by entering an interaction term between sex and each of the fixed-effect variables in the model and extracting the interaction coefficient of sex with species.

#### Sensitivity Analyses

In 3 separate analyses, we excluded 3 groups of participants: those in whom proton-pump inhibitors were measured in plasma, those with inflammatory bowel disease, and those treated with antibiotics during the year before the baseline visit. Furthermore, the analyses for CACS-associated species were repeated with the Shannon diversity index as a covariate to test whether findings were driven by global changes in the gut microbiota composition rather than changes in individual species. The associations from the main model were tested in an ordinal mixed model using CACS categories as the outcome (CACS 0, 1 to 100, 101 to 400, or >400) using Stata version 15.

#### Association Between CACS-Associated Species and Coronary Computed Tomography Angiography–Based Measurements and Carotid Plaques

We assessed the association of CACS-associated species with presence of any coronary atherosclerotic plaque or coronary stenosis >50% in at least 1 vessel in a series of logistic mixed regressions. The association between CACS-associated species and SIS, modified Duke index, and number of carotid vessels with atherosclerosis plaques were assessed with ordinal mixed regression models. Categories 4, 5, and 6 of the modified Duke index were merged because of a low number of observations. The logistic and ordinal mixed models were fitted using Stata version 15 and were adjusted for the main model covariates.

#### Clustering of Plasma Metabolites Associated With CACS-Associated Species

For each CACS-associated species, the 3 strongest associations with plasma metabolites were selected on the basis of their *P* value. For the resulting unique subset of 63 metabolites, hierarchical clustering was performed on the basis of the Euclidian distance on the partial Spearman correlation coefficients.

#### Associations Between CACS-Associated Species and Markers of Systemic Inflammation and Infection

A series of linear mixed models was fitted to assess the association between CACS-associated species (independent variable) and ln-transformed hsCRP, and ln-transformed counts of neutrophils and leukocytes, separately, as the dependent variable. The models were adjusted for the same covariates as the main model.

#### Associations Between Gut and Oral CACS-Associated Species and Between Oral CACS-Associated Species and Oral Health Phenotypes

To investigate the association between CACS-associated species in the gut and the corresponding species in saliva, MOS/MODS data were analyzed with a series of partial Spearman correlations from a mixed model on the basis of rank-transformed data (details in the Expanded Methods in the Supplemental Material). The models were adjusted for age, sex, and metagenomics extraction plate as fixed effects and family relatedness as a random effect. The oral species associated (*q*<0.05) with the corresponding CACS-associated gut species were assessed for association with 3 oral health phenotypes: filled surfaces (split by deciles in 10 categories), caries, and gingival inflammation. Filled surfaces and caries were assessed using ordinal mixed regressions using the ordinal version 2019.12-10 R package; gingival inflammation was assessed with a linear mixed model. These regression models were adjusted for age, sex, smoking, education, and Silness-Löe plaque index; as a marker of oral hygiene; and for activity in the hour immediately before the dental examination (eg, eating, brushing teeth, smoking) as fixed effects and family relatedness as a random effect.

## RESULTS

### Gut Microbiota Composition and Richness Are Associated With Subclinical Atherosclerosis and Attenuated by Adjustment for Lifestyle Factors, Diet, and Medication

**Figure 2.**
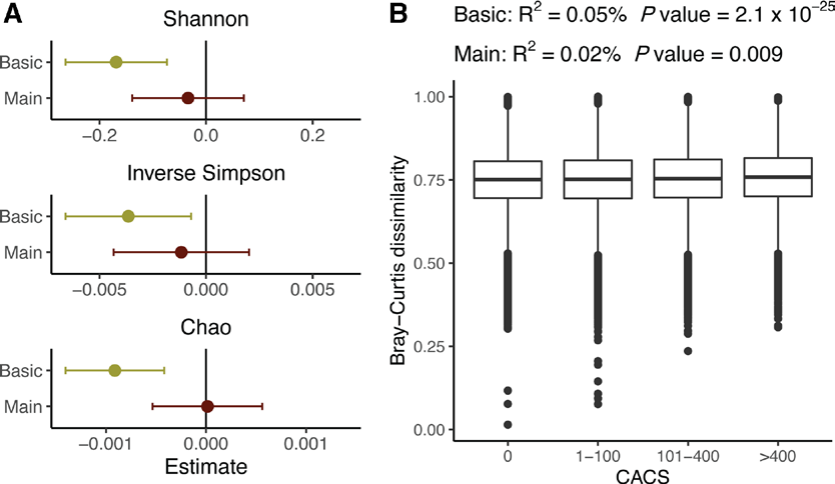
**Association of alpha and beta diversity with coronary artery calcium score. A**, The association of alpha diversity with coronary atherosclerosis was assessed by fitting linear mixed models with coronary artery calcium score (CACS) as the outcome and the alpha diversity indices as the exposure. The basic model (n=8972) was adjusted for age, sex, country of birth, and metagenomics extraction plate as fixed effects and family relatedness as random effect. The main model (n=8118) was adjusted further for smoking; physical activity; energy-adjusted intake of carbohydrate, protein, and fiber; systolic and diastolic blood pressure; total cholesterol; high-density lipoprotein and low-density lipoprotein cholesterol levels; triglycerides; body mass index; diabetes; and self-reported medication for dyslipidemia, hypertension, and diabetes as fixed effects. **B**, For beta diversity, a distance-based multivariate ANOVA of CACS as the independent variable was applied and the same fixed-effect adjustments were made without the random-effect adjustment. In the basic (n=8757) and the full model (n=7921), one participant for each family relatedness cluster was removed. Box plots represent the Bray-Curtis dissimilarity between participants with CACS=0 and the other CACS groups. For CACS=0, the boxplot represents the within-group dissimilarity.

The study included 8973 participants with no self- reported history of CVD. Among them, 40.3% had measurable coronary artery calcification and 5.4% manifested at least 1 stenosis with >50% occlusion (Table and Table S1). The Shannon diversity index—a measure of overall species richness and evenness within each sample—was inversely associated with CACS (n=8972, β=−0.17, *P*=5.1×10^−^^4^) in the basic model. The basic model was adjusted for age, sex, country of birth, and technical variables as fixed effects, and family relatedness as a random effect. However, the association was attenuated and nonsignificant in the main model with more extensive adjustment (n=8118, β=−0.03, *P*=0.53). The covariates primarily attenuating the association were triglycerides and body mass index, followed by medication, blood pressure traits, smoking, high-density lipoprotein cholesterol, and physical activity (Figure S3). Alternative alpha diversity indices were similarly associated with CACS (Figure 2A). Beta diversity—a measure of the amount of variation in species composition—differed among CACS values in both models, although the main model was attenuated (*r*^2^_basic_=0.0005; *P*_basic_=2×10^−25^ vs *r*^2^_main_=0.0002; *P*_main_=0.009) mainly by the same covariates that attenuated the relationship between alpha diversity and CACS (Figure 2B and Figure S3). These models were not adjusted for family relatedness, but only 1 of the participants in each family cluster was included instead (n_basic_=8757, n_main_=7921). These findings encouraged us to investigate species-specific associations.

**Table.**
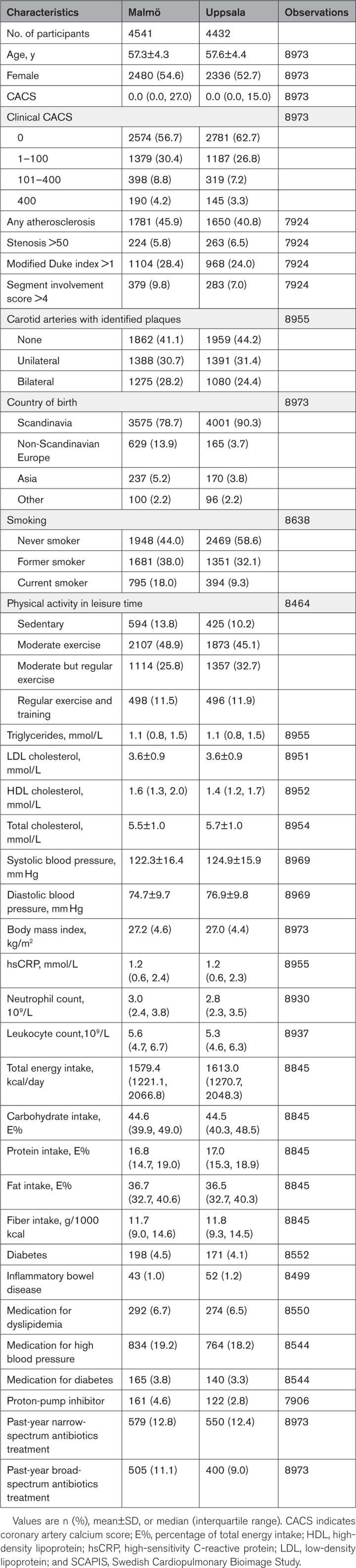
Descriptive Characteristics of Participants in SCAPIS With Metagenomics Data and No Previous Cardiovascular Disease Event

### Species Associated With Subclinical Atherosclerosis Are Enriched in Species From *Streptococcus* and *Oscillibacter* Genera

**Figure 3.**
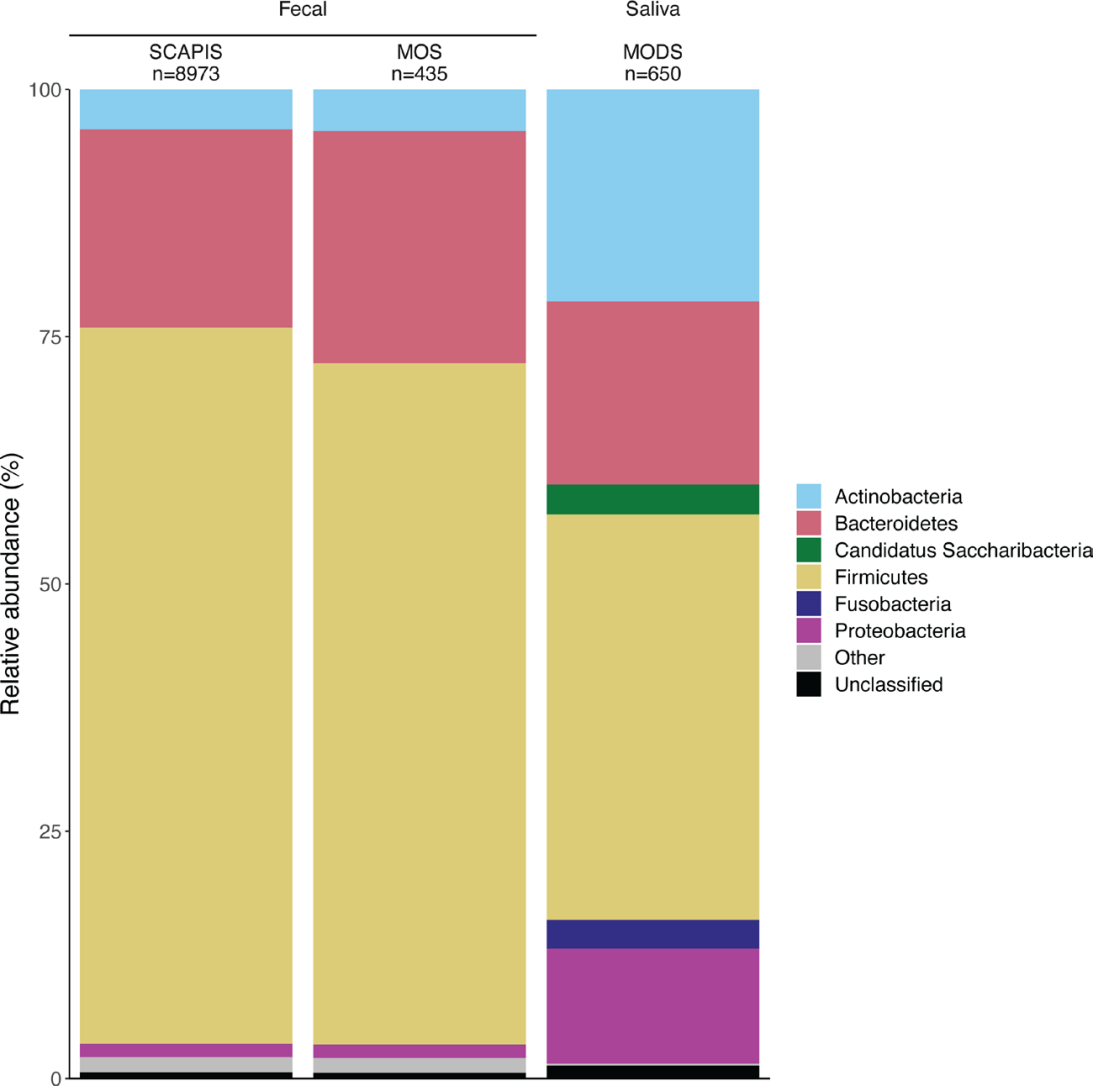
**Phylum-level taxonomic profiling of the fecal and saliva samples across the 3 data sets.** MODS indicates Malmö Offspring Dental Study; MOS, Malmö Offspring Study; and SCAPIS, Swedish Cardiopulmonary Bioimage Study.

The taxonomic profiling of fecal samples identified the prevalence of 1985 species, with 411 species having a prevalence <1%. The average number of species per sample was 325, with a range of 11 to 696. The dominant phyla were *Firmicutes* (72%) and *Bacteroidetes* (20%; Figure 3). In the basic model, including 8973 individuals, the relative abundance of 78 species (73 of which had a prevalence ≥1%) showed a positive association with CACS, whereas 29 species (all of which had a prevalence ≥1%) were negatively associated (Table S2). Taxon-set enrichment analysis revealed an overrepresentation of *Streptococcus* and *Oscillibacter* genera in the positive CACS associations (Table S3).

### Sixty-four Species Were Associated With CACS Independent of Cardiovascular Risk Factors

**Figure 4.**
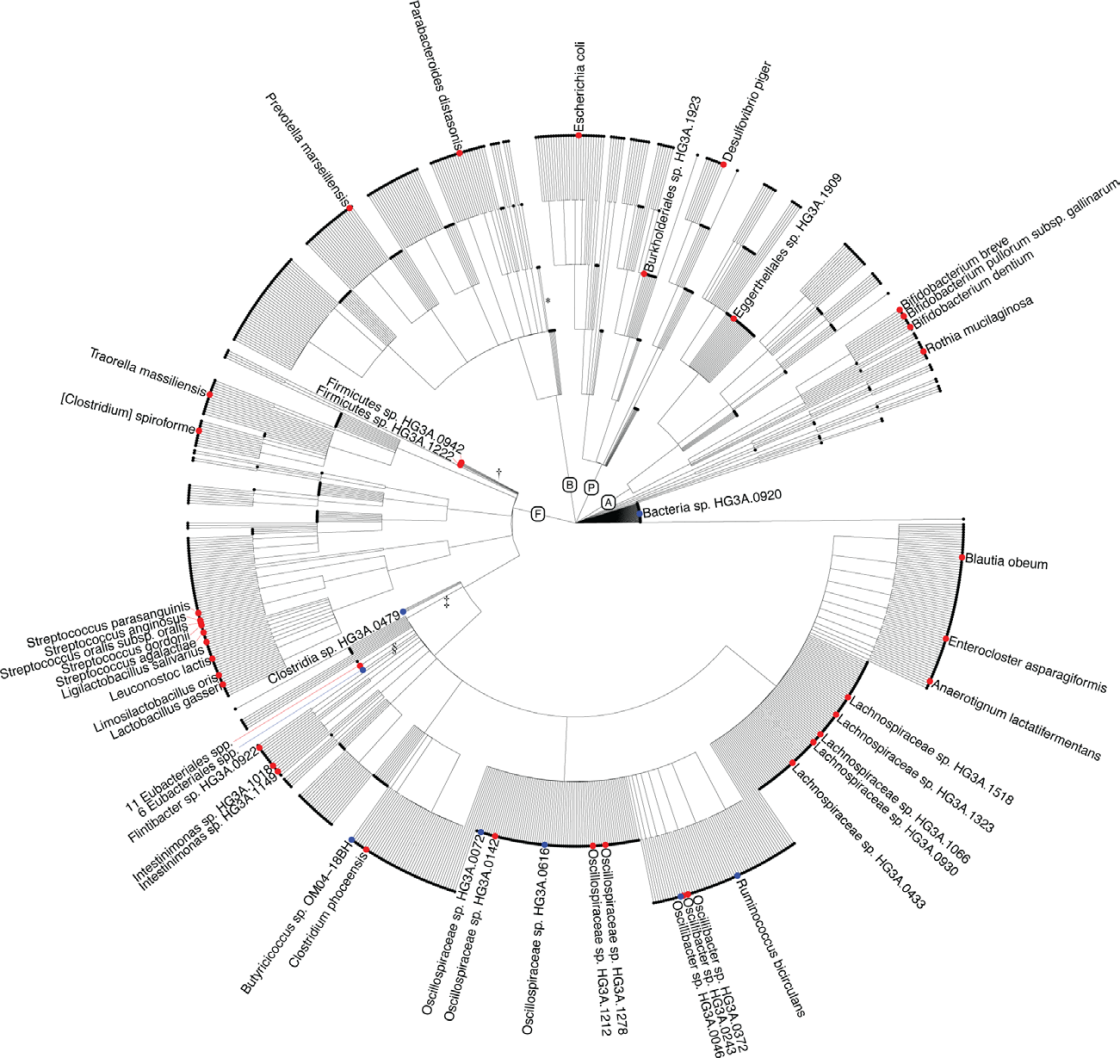
**Cladogram of species investigated in the study.** Circles represent metagenomic species annotated to the lowest taxonomic level. Dots indicate the species positively (red) and negatively (blue) associated with coronary artery calcium score (CACS) in the main model (false discovery rate *q*<0.05). The following unclassified non–CACS-associated metagenomics species were collapsed: *99 species with lowest annotation to the order *Bacteroidales*; †95 species with lowest annotation to phylum *Firmicutes*; ‡163 species with lowest annotation to the class *Clostridia*; and §651 species with lowest annotation to the order *Eubacteriales*. A indicates *Actinobacteria*; B, *Bacteroides*; F, *Firmicutes*; and P, *Proteobacteria*.

**Figure 5.**
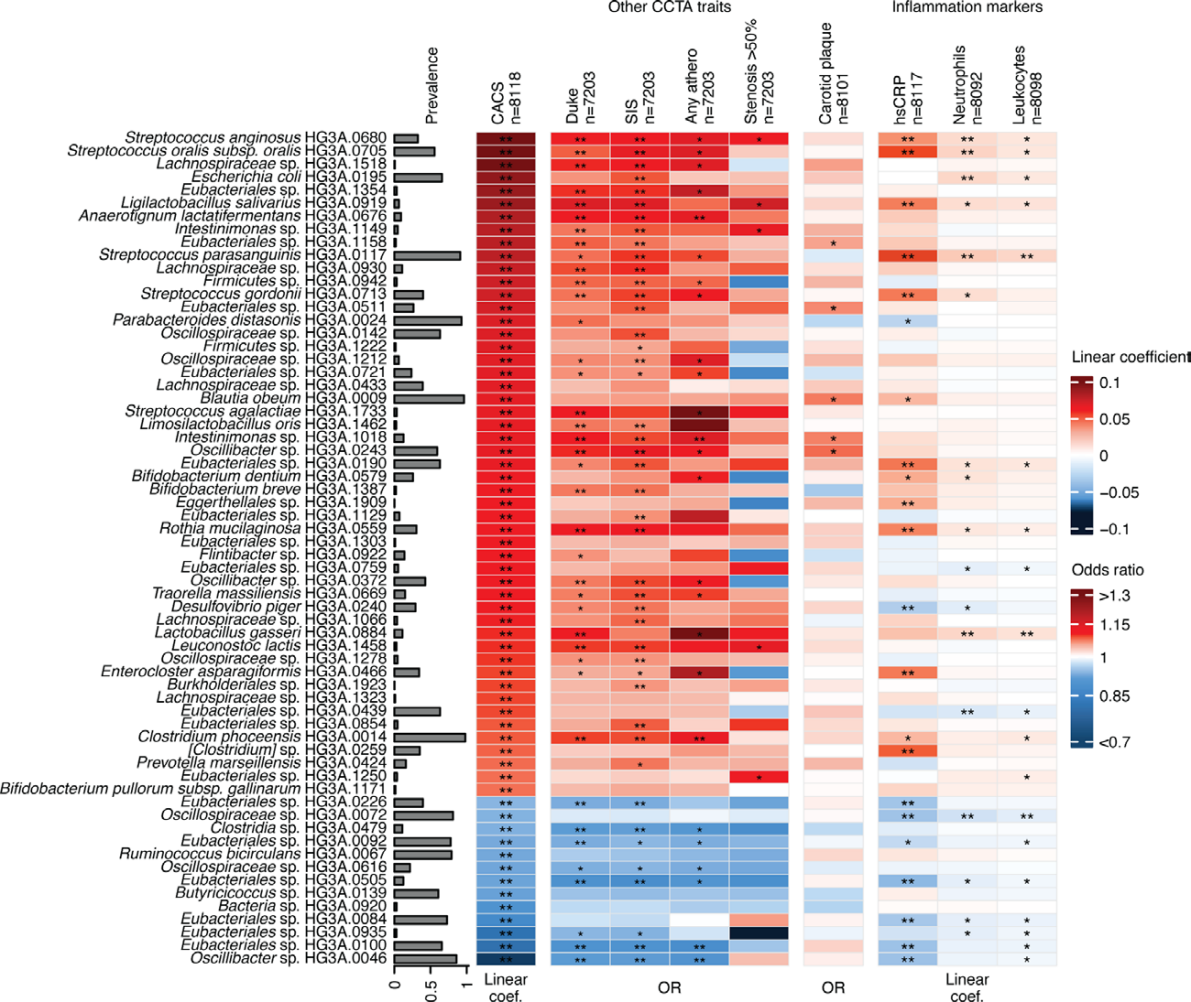
**Associations between coronary artery calcium score–associated gut species and alternate measurements of atherosclerosis and markers of inflammation and infection.** Heatmap showing the associations of the 64 coronary artery calcium score (CACS)–associated species (false discovery rate *q*<0.05) with CACS, other coronary computed tomography angiography atherosclerosis traits, carotid plaque, and inflammation biomarkers. Associations with CACS and with inflammatory markers high-sensitivity C-reactive protein and neutrophil and leukocyte counts are presented as linear regression coefficients after adjustment for the main model covariates (age; sex; country of birth; metagenomics extraction plate; smoking; physical activity; energy-adjusted carbohydrate, protein, and fiber intake; systolic and diastolic blood pressure; high-density lipoprotein, low-density lipoprotein, and total cholesterol levels; triglycerides; body mass index; diabetes; and self-reported medication for dyslipidemia, hypertension, and diabetes as fixed effects and first-degree family relatedness as a random effect). Associations with atherosclerosis traits (ie, modified Duke index, segment involvement score, any atherosclerosis, and ≥50% stenosis) and presence of carotid plaques are presented as odds ratio (OR) after adjustment for main model covariates. Associations with false discovery rate *q*<0.05 are marked with 2 asterisks (**) and those with *P*<0.05 are marked with 1 asterisk (*).

In the main model, which included 8118 individuals with complete data on covariates, 67 species remained associated with CACS (54 positively and 13 negatively; Figure 4). Covariates included age; sex; country of birth; metagenomics extraction plate; smoking; physical activity; energy-adjusted carbohydrate, protein, and fiber intake; blood pressure; lipid levels; diabetes; body mass index; self-reported cardiovascular medication; and family relatedness. However, the associations of *Peptoniphilus harei*, *Muribaculaceae* sp (metagenomic species identifier: HG3A.1967), and *Eubacteriales* sp (HG3A.0270) were deemed nonrobust, as the removal of single influential observations abolished the association (Table S4). Among the remaining 64 CACS-associated species, 51 had a positive association with CACS, whereas 13 had negative associations (Figures 4 and 5). The abundance of bacteria across CACS groups and the previously excluded SCAPIS participants with self-reported atherosclerotic CVD is shown in Figure S4. The lowest *P* values were observed for *Streptococcus anginosus*, *Streptococcus oralis* subsp *oralis*, *Escherichia coli*, *Eubacteriales* sp (HG3A.1354), and *Intestinimonas* sp (HG3A.1149), all positively associated with CACS. Other associated species included *Streptococcus parasanguinis*, *Streptococcus gordonii*, and *Streptococcus agalactiae*. Comparison of the main clinical characteristics in participants with high and low abundance of CACS-associated species (cutoff median) showed that those with higher abundance of the 2 most strongly associated species, *S* *anginosus* and *S* *oralis* subsp *oralis*, had in general more cardiovascular risk factors (Table S5). Furthermore, there were indications that the associations between *S* *agalactiae*, *Rothia mucilaginosa*, 2 *Eubacteriales* spp (HG3A.0511 and HG3A.0854), and an *Oscillibacter* sp (HG3A.0243) and CACS were modified by sex (*P*_interaction_<0.05; Table S4). After adjustment for multiple testing, the interaction terms were no longer significant.

### Sensitivity Analyses

The exclusion of individuals who used proton-pump inhibitors, had inflammatory bowel disease, or were treated with antibiotics in the previous 12 months before the baseline visit, as well as adjusting for Shannon diversity index showed largely similar effect estimates as the main model (Figure S5). Applying ordinal mixed models showed consistent effect direction and *P*<0.05 for 61 out of 64 species (Table S4). Overall, these findings indicate that the associations identified in the main analysis were reliable.

### Gut Metabolic Modules Related to Dissimilatory Nitrate Reduction, Anaerobic Fatty Acid β-Oxidation, and Amino Acid Degradation Are Positively Associated With Subclinical Atherosclerosis

In the basic model (n=8973), 36 GMMs were positively associated, whereas 4 had a negative association with CACS (Table S6). In the main model (n=8118), 11 GMMs were found to be positively associated with CACS, and none was negatively associated with CACS (Table S7). None of these associations was driven by an influential observation. These 11 GMMs are involved in various metabolic pathways, including amino acid degradation (eg, asparagine, proline, and valine degradation), anaerobic fatty acid β-oxidation, enzymatic reactions on lactate consumption, propionate production, and nitrogen metabolism.

### Other Atherosclerosis Measurements

Next, we assessed the association between CACS-associated species and 4 coronary computed tomography angiography measurements (presence of coronary atherosclerosis plaque, coronary stenosis >50%, modified Duke index, and SIS) and the number of carotid vessels with atherosclerotic plaque adjusted for the covariates in the main model. Twenty-five CACS-associated species were also associated with any coronary atherosclerotic plaque, 5 with coronary stenosis >50%, 39 with the modified Duke index, 44 with the SIS, and 5 with carotid plaque (*P*<0.05), all in the same direction as with CACS (Figure 5). The correlation among atherosclerosis measurements is shown in Figure S6.

### CACS-Associated Species Are Linked to Numerous Plasma Metabolites and Clustered Into Distinct Groups on the Basis of Their Strongest Associations

We assessed whether the 60 CACS-associated species available in the GUTSY Atlas^21^ describing species–metabolite associations were associated with plasma metabolites. All 60 species had at least 1 association with a plasma metabolite and there were 2500 associations (1478 positive and 1022 negative) between CACS-associated species and metabolites in total (Table S8). On the basis of their strongest associations with metabolites, the species clustered in distinct groups, with 1 group containing species commonly found in the oral microbiome^24^ (eg, all CACS-associated *Streptococcus* spp, *R* *mucilaginosa, Bifidobacterium dentium*, and *Ligilactobacillus salivarius*). Among others, this group was negatively associated with a set of uncharacterized molecules and with indole propionate, and positively associated with secondary bile acids and imidazole propionate. The remaining species were dominated by *Eubacteriales* spp, and included both CACS-positively and CACS-negatively associated species and had an inverse metabolic pattern compared with the oral microbiome set, with the exception of tobacco-related metabolites, which were positively associated with CACS-positively associated species and negatively associated with CACS-negatively associated species (Figure S7).

### Oral Species in the Gut Microbiota Are Associated With Markers of Systemic Inflammation and Infection

We assessed associations between CACS-associated species and plasma hsCRP (n=8117) and blood counts of leukocytes (n=8098) and neutrophils (n=8092) in linear mixed models. Of 54 species positively associated with CACS, 13 were also positively associated with hsCRP, 10 with leukocyte counts, and 11 with neutrophil counts (*P*<0.05). Species associated with all 3 markers were dominated by species common in the oral microbiome,^24^ including the streptococci species most strongly associated with CACS: *S* *anginosus*, *S* *oralis* subsp *oralis*, and *S* *parasanguinis.* Among the 13 species negatively associated with CACS, 6 were negatively associated with hsCRP, 6 with leukocyte counts, and 5 with neutrophil counts (Figure 5).

### CACS-Associated Species From Fecal Samples Correlate With Corresponding Species in Saliva

Several of the CACS-associated species are normally considered as oral species.^24^ We therefore investigated the correlation between the abundance of CACS-associated species in the fecal and saliva samples from the 435 participants in MODS (Table S9) who underwent a thorough dental examination within 4 to 12 months after fecal sampling in MOS. Six of the CACS-associated species were identified in the saliva samples, of which 5 (*S* *anginosus, S* *parasanguinis*, *S* *gordonii*, *R* *mucilaginosa*, and *B* *dentium*) were positively associated with the abundance of the corresponding species in the fecal sample (ρ=0.15–0.46), whereas *S* *oralis* subsp *oralis* was not (Table S10). In up to 639 participants from MODS, 3 species (*S* *anginosus, S* *parasanguinis*, and *R* *mucilaginosa*) were positively associated with both caries and filled surfaces, and 3 (*S* *anginosus, S* *gordonii*, and *B* *dentium*) with gingivitis (Table S11).

## DISCUSSION

Gut bacteria have been proposed to affect the development and progression of atherosclerosis through infections local or distal to the atherosclerotic plaque or through production of atherogenic metabolites.^2^ The association of gut microbiota with coronary atherosclerosis has previously been studied only in symptomatic patients, who are often under treatment, resulting in high risk of bias as medication may result in large shifts of the composition. Here, detailed image-based measurements of coronary artery atherosclerosis and deep characterization of the gut microbiome by shotgun metagenomics of individuals without earlier cardiovascular disease in the large population-based SCAPIS cohort were used to identify 64 species associated with CACS independent of risk factors. Several of these species were associated with circulating markers of inflammation and infection, and with the corresponding bacteria species in saliva samples, which were in turn associated with worse oral health. Thus, studies are merited to continue to investigate the role of these 64 species in atherogenesis.

The *Streptococcus* genus was associated with CACS, consistent with observations in previous case–control studies of symptomatic atherosclerotic cardiovascular disease.^6,7,25^ Specifically, *S* *anginosus*, *S* *oralis* subsp *oralis*, *S* *parasanguinis*, *S* *gordonii*, and *S* *agalactiae* were associated with coronary atherosclerosis. Current knowledge of the involvement of these bacteria in cardiovascular pathologies is summarized in Table S12. These species all belong to the VGS,^26^ except for *S* *agalactiae* (a β-hemolytic non-VGS). VGS can enter the bloodstream through mucosal barrier injuries from daily dental care activities and dental procedures, and could also pass the gut barrier when injured.^27^ VGS can infect the valves and the coronary vessels, accounting for 20% of infective endocarditis cases.^4,9,28^ VGS species initiate and contribute to biofilm formations, which enhance bacterial survival and have been found in atherosclerotic lesions with unclear significance.^29^ Moreover, in model systems, VGS can invade human aortic endothelial cells and stimulate atherosclerosis-related proinflammatory cytokines.^30^ Animal studies suggest a causal link between *Streptococcus* spp and atherogenesis.^31,32^ In our study, the abundance of CACS-associated *Streptococcus* spp in the gut associated strongly and positively with hsCRP, leukocytosis, and neutrophilia, which could have been triggered by low-grade bacteremia.

We assessed the association of the CACS-associated species with other measures of coronary atherosclerosis from the coronary computed tomography angiography as well as with carotid atherosclerosis measured by ultrasound. In general, we found high agreement of associations of species with CACS, modified Duke index, any atherosclerosis, and SIS. However, the associations with having an occlusion >50% of at least 1 vessel were weaker, perhaps because this was rather rare in our cohort, with low power. Likewise, only a few of the species were associated with carotid plaque, perhaps because the measurement of carotid plaque was not very detailed or because associations differ in different vascular beds.

The gut microbiota composition might also contribute to atherogenesis through alteration of the host metabolism. In the current study, all CACS-associated species were associated with at least 1 plasma metabolite and clustered in distinct groups on the basis of their associations with the metabolome. One group, which contained all the species commonly found in saliva, was positively associated with several microbiota-derived metabolites such as primary and secondary bile acids, and with omeprazole and metformin. Furthermore, this group was negatively associated with the microbially derived tryptophan metabolite indole propionate, a metabolite that has been found inversely associated with atherosclerotic coronary disease in humans and reduced progression of atherosclerosis in mice.^33^ Bacteria of this group were also found positively associated with imidazole propionate, a microbially derived metabolite from histidine, reported to impair glucose metabolism.^34^ Several tobacco metabolites (ie, 3-hydroxycotinine glucuronide, cotinine N-oxide, and norcotinine) were positively associated with species positively associated with CACS and negatively associated with species negatively associated with CACS. Together, these findings suggest an interplay between CACS-associated species and microbial metabolite production, drug intake, and smoking behavior.

Another aspect of the microbiome is the functional metabolic potential, which can be summarized into metabolic modules on the basis of known functions of bacterial genes.^20^ We found 11 modules positively associated with CACS, with the strongest associations noted for dissimilatory nitrate reduction and anaerobic fatty acid β-oxidation. Dietary nitrate is absorbed in the intestines and excreted back into the gastrointestinal tract by the salivary glands at high concentrations, forming the so-called entero-salivary circulation of nitrate. Denitrifying oral bacteria are important for converting nitrate (NO_3_^−^) into nitrite (NO_2_^−^), which can be further converted into nitric oxide (NO) in the acidic pH of the stomach. The generation of NO contributes to gastric protection by increasing blood flow and mucus thickness.^35^ The nitrate and nitrite that are not converted to NO in the stomach are absorbed into the bloodstream and tissues, where they might act as reservoir for NO, which appears to be important for vasodilation under hypoxia^36^ and modulation of mitochondrial respiration.^37^ However, the dissimilatory nitrate reduction pathway converts nitrate to ammonia, and having an increased activity of this pathway in the colon could potentially inhibit the potential positive cardiovascular effects of nitrate. We found that genes related to anaerobic fermentation of fatty acids were associated with CACS. Such genes are rather uncommon in the gut microbiota and the genetic background of oxidation of fatty acids under anaerobic conditions is not well-characterized.^20^ We found an association of 5 amino acid breakdown modules with CACS. This could be driven by differences in diet not captured by our covariates.

Our study has some limitations. First, although our cohort is at least 7 times larger than those analyzed previously, few participants had high levels of subclinical atherosclerosis, reducing statistical power. Second, microbial composition can vary extensively throughout the gastrointestinal tract. Fecal samples contain microbial populations from the distal colon, and less so from other sites, such as the small intestine. Therefore, we could not identify associations of species that are not well represented in fecal samples. Third, our study does not take into consideration the different interactions among bacterial species such as synergistic effects in the relationship with coronary atherosclerosis. Fourth, the cross-sectional study design limits causal inference. Last, our data on antibiotic treatment do not capture antibiotics provided in inpatient care; however, in this age group, we do not expect a large number of hospital-treated patients. In future studies, different causal inference methods should be used to determine whether the identified species are causally related to atherosclerosis development. By combining data from a large population-based cohort study and imaging to evaluate subclinical coronary atherosclerosis, we found that the abundance of several species in the gut was associated with coronary atherosclerosis, biomarkers of inflammation, and their oral counterparts. If causal, these species might contribute to atherogenesis by direct infection or by altering host metabolism. Future studies will show whether these species can be used as potential biomarkers or treatment targets.

## ARTICLE INFORMATION

### Sources of Funding

The main financial support for the study was provided by the European Research Council (ERC-2018-STG-801965 [Dr Fall], ERC-CoG-2014-649021 [Dr Orho-Melander], and ERC-STG-2015-679242 [Dr Smith]). Funding was also provided by the Swedish Research Council (VR, 2019-01471 [Dr Fall], 2018-02784 [Dr Orho-Melander], 2018-02837 [Dr Orho-Melander], 2019-01015 [Dr Ärnlöv], 2020-00243 [Dr Ärnlöv], 2019-01236 [Dr Engström], 2017-02554 [Dr Smith], and 2022-01460 [Dr Ahmad], and EXODIAB 2009-1039 [Dr Orho-Melander]); the Swedish Heart-Lung Foundation (Hjärt-Lungfonden 20190505 [Dr Fall], 20200711 [Dr Orho-Melander], 20180343 [Dr Ärnlöv], 2019-0526 [Dr Smith], and 20200173 [Dr Engström]); an ALF governmental grant (2018-0148 [Dr Orho-Melander]); the Novo Nordisk Foundation (NNF20OC0063886 [Dr Orho-Melander]); the Swedish Diabetes Foundation (DIA 2018-375 [Dr Orho-Melander]); the Swedish Foundation for Strategic Research (LUDC-IRC 15-0067 [Dr Orho-Melander]); Formas (2020-00989 [Dr Ahmad]); Göran Gustafsson Foundation (2016 [Dr Fall]); and Axel and Signe Lagerman’s Foundation (Dr Fall). SCAPIS (Swedish Cardiopulmonary Bioimage Study) was funded mainly by the Swedish Heart-Lung Foundation. The study was also funded by the Knut and Alice Wallenberg Foundation, the Swedish Research Council, VINNOVA (Sweden’s innovation agency), the University of Gothenburg and Sahlgrenska University Hospital, Karolinska Institutet and Stockholm County Council, Linköping University and University Hospital, Lund University and Skåne University Hospital, Umeå University and University Hospital, and Uppsala University and University Hospital. MODS (Malmö Offspring Dental Study) was funded mainly by Oral Health Related Research by Region Skåne (OFRS 422361, OFRS 512951, OFRS 567711, OFRS 655561, OFRS 752071, OFRS 853031, OFRS 931171, and OFRS968144). MOS (Malmö Offspring Study) was funded by the Swedish Research Council (VR, 521-2013-2756 [Dr Nilsson]), the Swedish Heart and Lung Foundation (Hjärt-Lungfonden 20150427 [Dr Nilsson]), and an ALF grant from the local Region Skåne County Council (Dr Nilsson). Additional funding was provided by Ernhold Lundströms Stiftelse (Dr Brunkwall). The computations and data handling were enabled by resources in project sens2019512 provided by the National Academic Infrastructure for Supercomputing in Sweden and the Swedish National Infrastructure for Computing at Uppsala Multidisciplinary Center for Advanced Computational Science, partially funded by the Swedish Research Council through grant agreements 2022-06725 and 2018-05973.

### Disclosures

S. Pita, N. Nielsen, and Drs Eklund, Holm, and Nielsen are employees of Clinical Microbiomics A/S, where samples were processed and DNA extraction and estimations of relative abundance of the metagenomics species were done. Dr Ärnlöv has received lecture fees from Novartis and AstraZeneca and has served on advisory boards for AstraZeneca and Boehringer Ingelheim, all unrelated to the article. Dr Nilsson has received lecture fees from Novartis, Novo Nordisk, Amgen, and Boehringer Ingelheim. The other authors declare no competing interests.

### Supplemental Material

Expanded Methods

Tables S1–S12

Figures S1–S7

References 38–45

## Supplementary Material


