# Supplementary material for: Streptococcus Species Abundance in the Gut Is Linked to Subclinical Coronary Atherosclerosis in 8973 Participants From the SCAPIS Cohort
Source: Circulation. 2023 Jul 12;148(6):459–72. doi: 10.1161/CIRCULATIONAHA.123.063914 (PMC10399955; doi:10.1161/CIRCULATIONAHA.123.063914)
Supplement: Supplementary file 1 [file cir-148-459-s001.pdf]

SUPPLEMENTAL MATERIAL

Autoimmune Atrial Fibrillation

EXPANDED METHODS

Study population

The present study was conducted using biospecimens from Discovery Life Sciences Biobank (Discovery Life Sciences, Inc., USA) as well as from the Montreal Heart Institute hospital cohort. Plasma samples were selected based on the International Classification of Diseases 10th Edition (ICD-10) code. Patients with code I48.0 (paroxysmal AF), I48.1 (persistent AF), I48.2 (permanent AF) or I48.91 (unspecified AF) were included in the AF group if they presented no conditions related to compromised immune system (ICD-10 codes D80-D90), cardiovascular diseases (I00-I97, with the exception of arterial hypertension (I10)) or congenital cardiac malformations (Q20-Q28), neoplasm (C00-D49) and thyroid disease (E00-E07). The Control group consisted of age- and sex-matched healthy individuals, i.e. with no ICD-10 codes. The Montreal Heart Institute hospital cohort provided patients assigned to the Pre-AF group: patients with the same exclusion criteria as for the AF group, but presenting a first diagnosis of AF at a timepoint after study inclusion.

Human Induced Pluripotent Stem Cell-Derived Atrial Cardiomyocytes

Human induced pluripotent stem cell-derived atrial cardiomyocytes (hiPSC-aCMCs) were custom manufactured and provided by Ncardia BV (Leiden, The Netherlands). Cells were derived from renal epithelial cells extracted from the urine of a female healthy volunteer. Using proprietary differentiation protocol, cells were differentiated into hiPSC-aCMCs. The atrial phenotype was ascertained by Ncardia through biomarker analysis (FACS, immunofluorescence) and functional testing with multielectrode assay (MEA). 86% of cells presented TNNT2, while 52% of TNNT2-positive population expressed Coup-TFII (8 days post-thaw). Furthermore, the addition of Ivabradine at ≥ 3 µM decreased the beating rate 30 min

after treatment (10 days post-thaw, data not shown). HiPSC-aCMCs were cultured according to Ncardia's guidelines. Briefly, Petri dishes were coated with Corning Matrigel (VWR, Dietikon, Switzerland) diluted 1:100 in DMEM/F-12 (STEMCELL Technologies, Cologne, Germany). Cryovials of hiPS-aCMCs were thawed and the cell suspension immersed in Pluricyte® Cardiomyocyte Medium in a drop-wise manner. Following centrifugation (250 g for 3 min), cells were resuspended in Pluricyte® Cardiomyocyte Medium before plating on Matrigel-coated Petri dishes at a density of 250'000 cells per cm^2. hiPSC-aCMCs were placed in an incubator (37°C, 5% CO_2) and culture medium changed every 48h.

Enzyme-linked Immunosorbent Assay

Blood samples from all mice was collected via venipuncture on day 28. To detect the presence of anti-K_{ir}3.4 IgG in the serum, standard ELISA was performed by BIOTEM (Apprieu, France). In brief, K_{ir}3.4 target peptide (2 µg/mL 1xPBS) was immobilized onto microtiter plates (overnight at room temperature (RT)). After washing steps with 1xPBS/0.05% Tween20, blocking buffer (1xPBS/2.5% dried skimmed milk) was used to block remaining unspecific binding sites for 1h at RT. Plates were washed and serially diluted serum samples (in 1xPBS/0.05% Tween20/0.5% BSA) added to the wells (2h at RT). Peroxidase AffiniPure F(ab')$_2$ Fragment goat anti-mouse IgG (Fcγ fragment specific from Jackson ImmunoResearch Europe Ltd, UK) was used as secondary antibody (1:5000 in 1xPBS/0.05% Tween20/0.5% BSA, 1h at RT). Wells were washed and incubated with TMB substrate prior to stopping the reaction with 0.1M H_2SO_4. Plates were then read for absorbance at 450 nm. Titers were defined as the serum dilution corresponding to half the maximal absorbance obtained.

Supplemental Table S1. Patient data. Statistical significance was determined by one-way ANOVA (age) and Chi-square test (sex).

	AF	Control	Pre-AF	P value
Patients, n	37	37	14	
Age, mean ± SD, y	69.8 ± 16.3	69.8 ± 16.4	67.0 ± 7.5	0.822
Sex, %				
Male	54.1	54.1	50.0	0.759
Female	45.9	45.9	50.0	0.759
Cardiovascular risk factors				
Arterial hypertension, %	27.0	0	35.7	
Diabetes mellitus, %	18.9	0	0	
Dyslipidemia, %	10.8	0	35.7	

Supplemental Table S2. Peptide Microarray data summary based on the corrected intensity values of all 88 tested human plasma samples.

S2.xlsx File.

Supplemental Table S3. Clinical characteristics and autoantibody status of study population.

Subject#	Group	Age (years)	Sex	Risk factors	Autoantibody status
1	Control	86	M		
2	Control	68	M		
3	Control	87	F		
4	Control	70	M		
5	Control	82	F		
6	Control	91	F		
7	Control	72	M		
8	Control	60	M		
9	Control	64	M		
10	Control	37	M		
11	Control	60	M		
12	Control	61	M		
13	Control	64	F		
14	Control	56	M		
15	Control	54	M		

16	Control	32	M		
17	Control	86	F		
18	Control	84	F		
19	Control	82	F		
20	Control	70	F		
21	Control	87	M		
22	Control	72	M		
23	Control	27	F		
24	Control	81	F		
25	Control	89	M		
26	Control	69	F		
27	Control	93	M		
28	Control	79	F		
29	Control	37	M		
30	Control	71	M		
31	Control	70	F		
32	Control	65	F		
33	Control	74	F		
34	Control	63	M		
35	Control	86	F		
36	Control	86	F		
37	Control	68	M		
38	AF	81	F		
39	AF	72	M	HT	
40	AF	69	M		
41	AF	72	M	HT	
42	AF	71	M		
43	AF	91	F		
44	AF	27	F	HT, DM, OB	
45	AF	64	M		
46	AF	60	M	DM	
47	AF	56	M		
48	AF	68	M		
49	AF	65	F	HT	Kir3.4^{+}
50	AF	70	F		
51	AF	86	F		
52	AF	81	F		
53	AF	86	F		
54	AF	74	F		
55	AF	38	M		
56	AF	36	M	HT, HL	Kir3.4^{+}
57	AF	64	M	HT, HL	
58	AF	87	M	HT, HL	
59	AF	84	F		Kir3.4^{+}
60	AF	64	F	DM	Kir3.4^{+}
61	AF	71	F		
62	AF	68	F		
63	AF	88	M		
64	AF	32	M		
65	AF	90	M		
66	AF	85	F	HT, DM	Kir3.4^{+}

67	AF	62	M	DM	
68	AF	79	F		
69	AF	54	M		$Kir3.4^+$
70	AF	72	M	HT	
71	AF	60	M		
72	AF	89	F	DM	
73	AF	85	M		
74	AF	81	F	HT, HL, DM	
75	Pre-AF	60	M	AL	
76	Pre-AF	54	F		$Kir3.4^+$
77	Pre-AF	74	F	HT, HL, OB	$Kir3.4^+$
78	Pre-AF	70	M	HL, OB	
79	Pre-AF	70	M	HT, OB	$Kir3.4^+$
80	Pre-AF	68	M	HT, HL, OB	$Kir3.4^+$
81	Pre-AF	55	M	OB	
82	Pre-AF	66	F	HL, OB	
83	Pre-AF	80	F		
84	Pre-AF	73	F		
85	Pre-AF	65	F	HT, HL, OB	
86	Pre-AF	77	M	HT, HL, OB	
87	Pre-AF	65	F	HT, OB	$Kir3.4^+$
88	Pre-AF	61	M	HT, HL, DM, OB	

Risk factors refer to modifiable AF risk factors: AL indicates alcohol consumption ≥ 1.2 unit/day; DM, type 2 diabetes mellitus; HL, hyperlipidemia; HT, arterial hypertension; and OB, obesity with BMI ≥ 25kg/m^2.

Supplemental Table S4. Action potential characteristics of hiPSC-CMC ± Carbachol ± K_{ir}3.4 autoantibody. Statistical significance was determined using one-way ANOVA followed by Tukey's multiple comparisons test.

	Control (n=6)	Carbachol (n=8)	Anti-K_{ir}3.4 IgG (n=8)	Anti-K_{ir}3.4 IgG + Carbachol (n=8)	P value
MDP, mean ± SEM, mV	-78.9 ± 2.0	-69.8 ± 3.4	-71.0 ± 2.8	-69.1 ± 2.7	0.051
APA, mean ± SEM, mV	105.9 ±3.7	92.9 ± 4.9	88.4 ± 4.9	85.4 ± 5.1	0.046
APD_{90}, mean ± SEM, ms	390.2 ± 50.5	307.2 ± 24.0	261.5 ± 17.5	203.2 ± 8.3	<0.001
Beating rate, mean ± SEM, bpm	37.5 ± 4.4	36.0 ± 3.7	46.3 ± 2.3	56.5 ± 6.5	0.018

MDP indicates maximum diastolic potential; APA, action potential amplitude; and APD_{90}, Action potential duration at 90% repolarization.

Supplemental Table S5. Electrophysiological parameters based on ECG and electrophysiological study (EPS). Statistical significance was determined using Student's t test (PR, QRS, QT, QTc, JTc; SNRT, cSNRT, AERP, AVERP) and Mann-Whitney U test (RR, JT, WCL).

	Sham-immunized mice (n=14)	K_{ir}3.4-immunized mice (n=10)	P value
ECG			
RR interval, mean ± SD, ms	145.5 ± 23.2	142.1 ± 11.0	0.656
PR interval, mean ± SD, ms	36.9 ± 4.9	35.4 ± 3.9	0.439
QRS duration, mean ± SD, ms	9.8 ± 1.7	9.6 ± 1.2	0.728
JT interval, mean ± SD, ms	46.4 ± 9.0	47.1 ± 6.1	0.931
JTc interval, mean ± SD, ms	38.6 ± 7.1	39.6 ± 5.4	0.702
QT interval, mean ± SD, ms	55.7 ± 9.7	56.7 ± 6.3	0.768
QTc interval, mean ± SD, ms	46.4 ± 8.2	47.6 ± 5.5	0.683
EPS			
SNRT, mean ± SD, ms	188.4 ± 28.2	190.7 ± 46.0	0.882
cSNRT, mean ± SD, ms	55.6 ± 22.7	53.3 ± 30.9	0.840
WCL, mean ± SD, ms	71.4 ± 7.7	71.1 ± 3.3	0.535
AVERP, mean ± SD, ms	60.4 ± 7.8	62.2 ± 5.8	0.560
AERP, mean ± SD, ms	47.3 ± 7.8	35.4 ± 7.7	0.001

SNRT indicates sinus node recovery time; cSNRT, corrected sinus node recovery time; WCL, Wenckebach cycle length; AVERP, atrioventricular effective refractory period; and AERP, atrial effective refractory period.

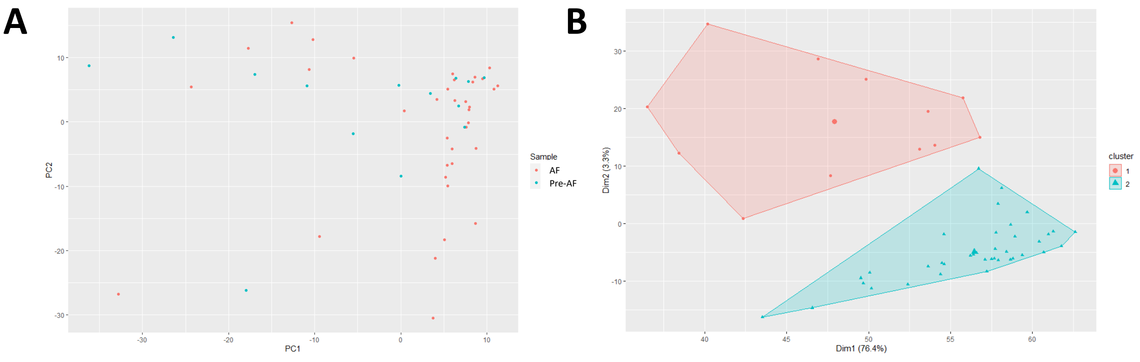

PCA Plot

K-means Cluster Plot

Supplemental Figure S1. Principal component analysis plot and k-means clustering of autoantibody profiles. Analysis was based on background-corrected median intensity data, which was further log-transformed for better visualization. Data points represented all values generated from a plasma sample (n=51). **A**, The principal component analysis (PCA) did not show any particular clustering of AF (n=37) or Pre-AF (n=14) samples. The sample distribution appeared to be rather heterogeneous. **B**, In a second step, we used k-means clustering algorithm to partition the dataset into a set of k groups. The algorithm classified the 2 groups with Euclidean distance. The cluster assignment steps are iteratively repeated until convergence is achieved. According to k-means clustering, there was also no clear separation of AF and Pre-AF samples into the two cluster assignments (1 and 2).

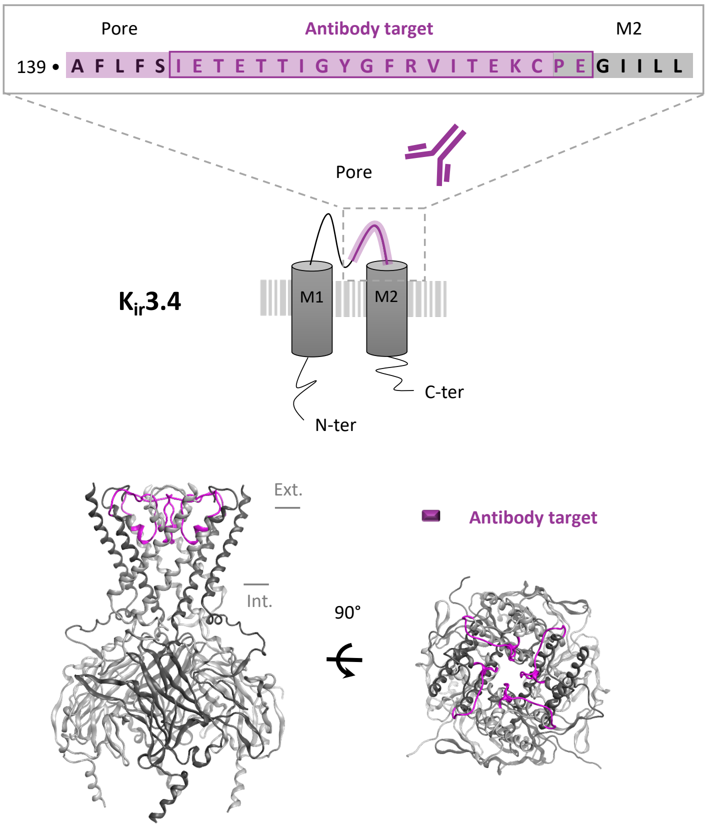

Supplemental Figure S2. Anti-K$_{ir}$3.4 IgG target epitope. A, Sequence alignment of the anti-K$_{ir}$3.4 IgG target (framed in purple) on the K$_{ir}$3.4 protein close to the pore domain. **B**, 3D-structural representation of the human K$_{ir}$3.4 channel. The target epitope is highlighted in purple. Molecular graphics were rendered using Molecular Operating Environment (MOE software version 2020.09, Chemical Computing Group, Canada) based on the human K$_{ir}$3.2 structure (PDB 3SYC) as a homology model, adapted to the human amino acid sequence of K$_{ir}$3.4 (NP_001341098.1). Ext. indicates extracellular; Int., intracellular; M1, transmembrane segment 1; and M2, transmembrane segment 2.
