# Supplementary figures and images for: Streptococcus Species Abundance in the Gut Is Linked to Subclinical Coronary Atherosclerosis in 8973 Participants From the SCAPIS Cohort
Source: Circulation. 2023 Jul 12;148(6):459–72. doi: 10.1161/CIRCULATIONAHA.123.063914 (PMC10399955; doi:10.1161/CIRCULATIONAHA.123.063914)

**A**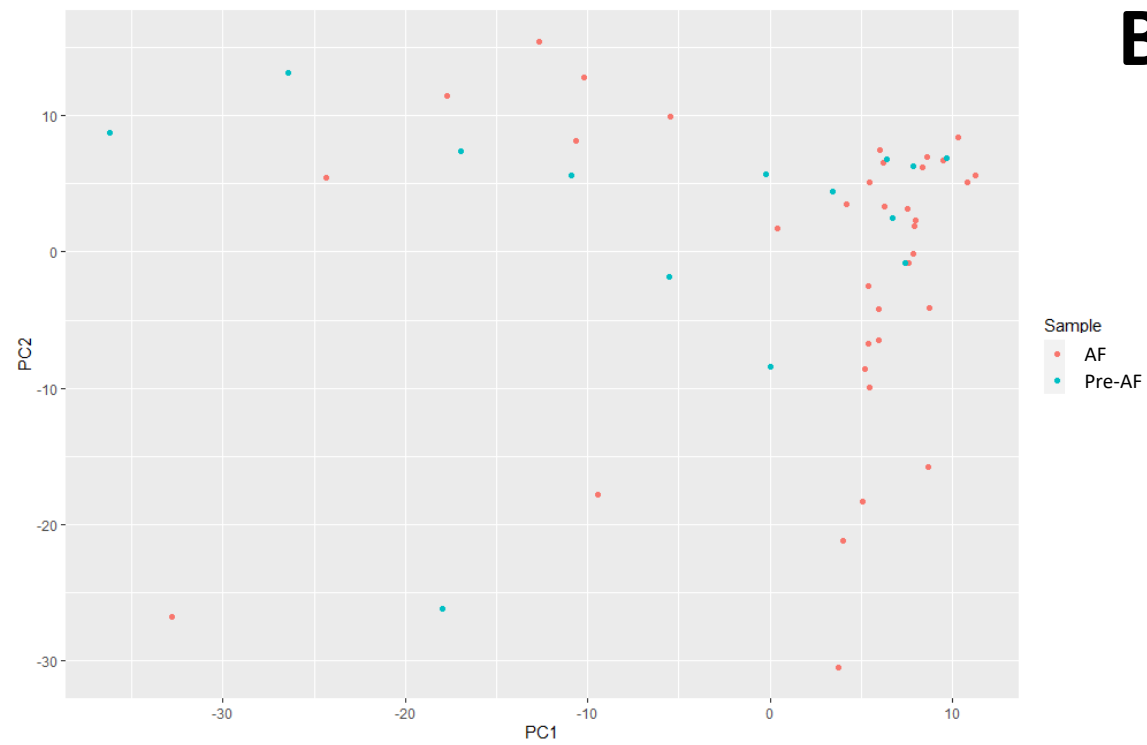**PCA Plot****B**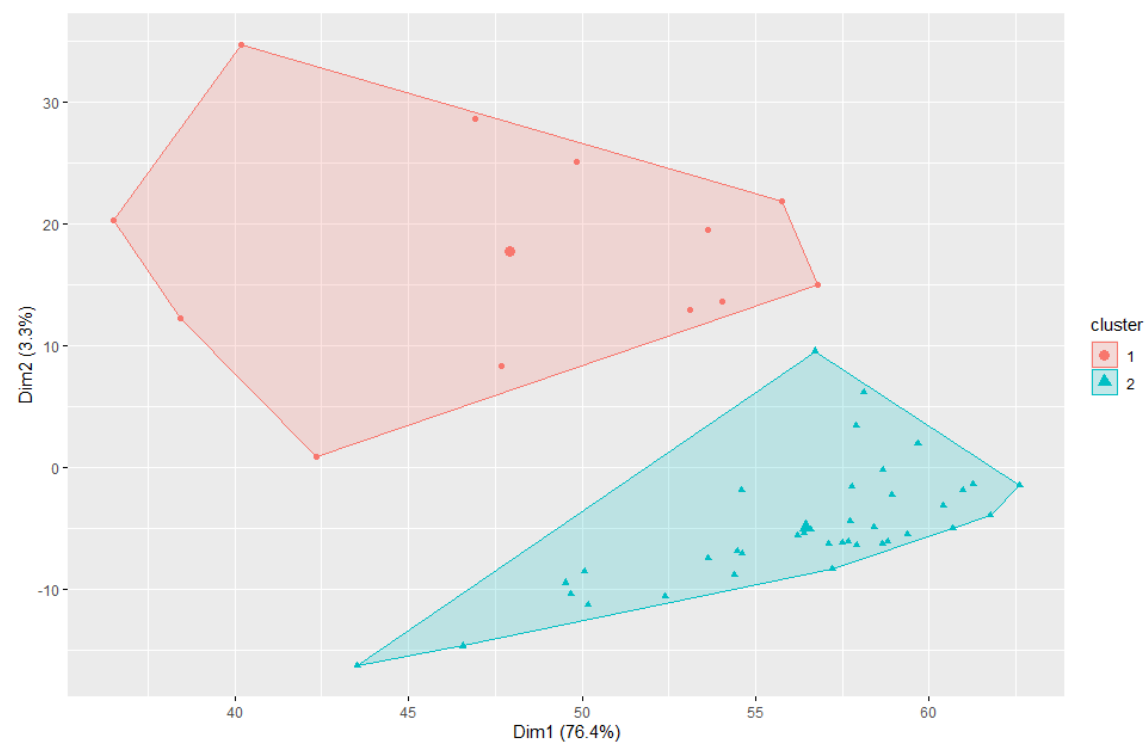**K-means Cluster Plot**

Supplement: Supplementary file 2 [file cir-148-459-s002.pdf]

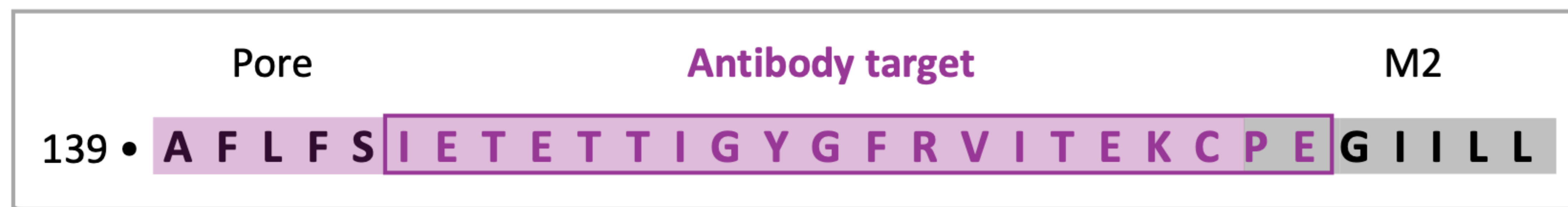

K<sub>ir</sub>3.4

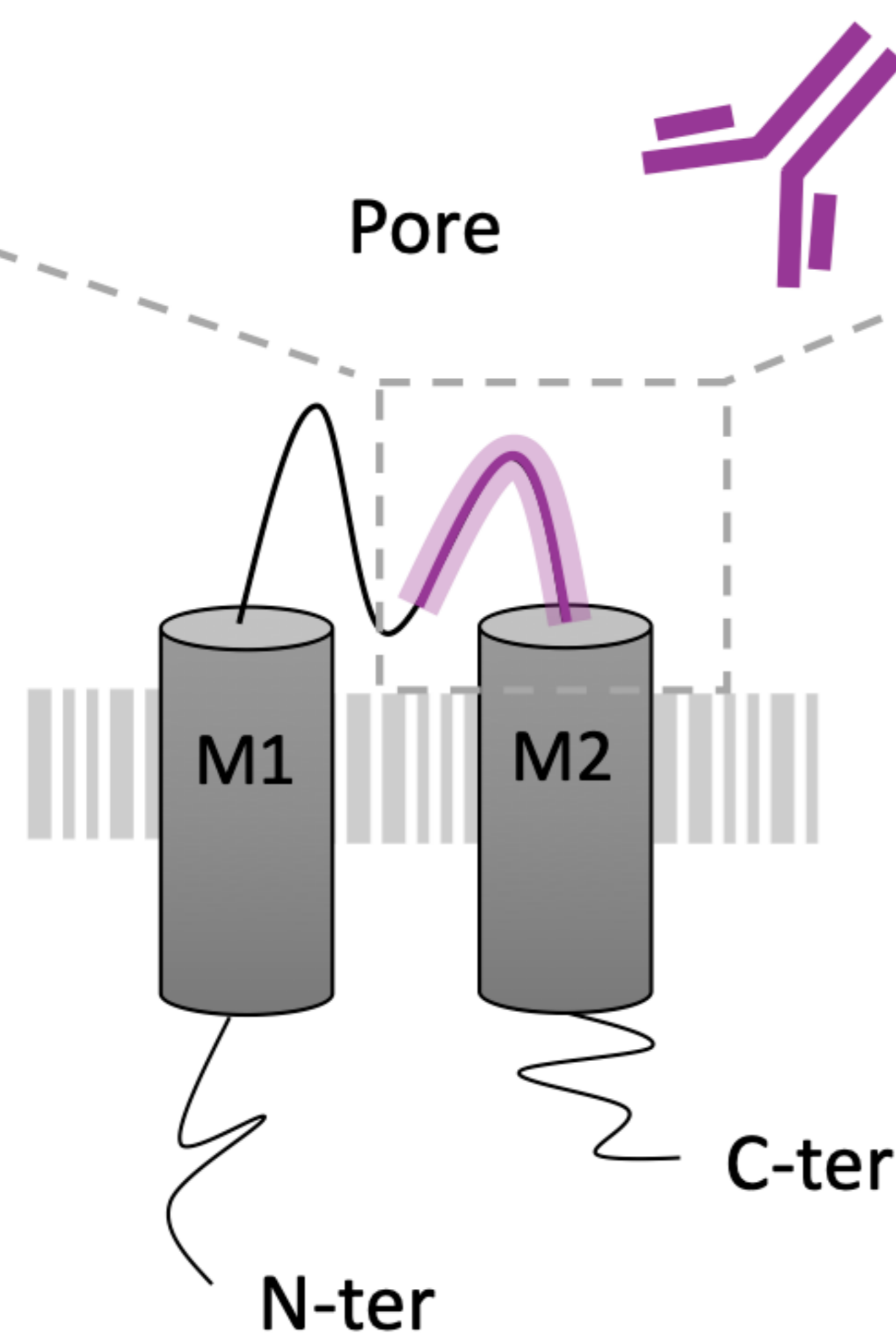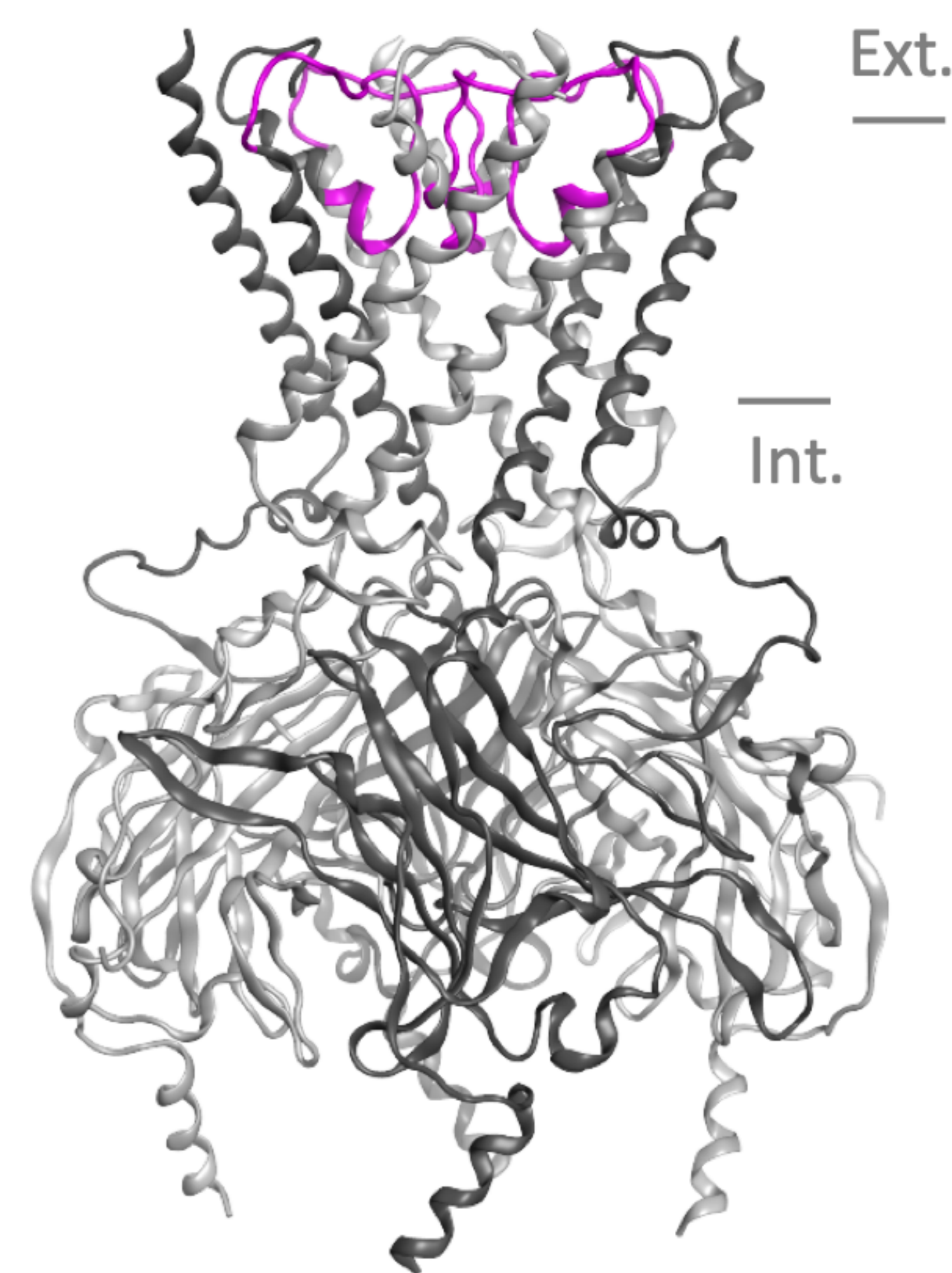

90°

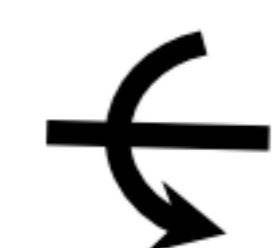

Antibody target

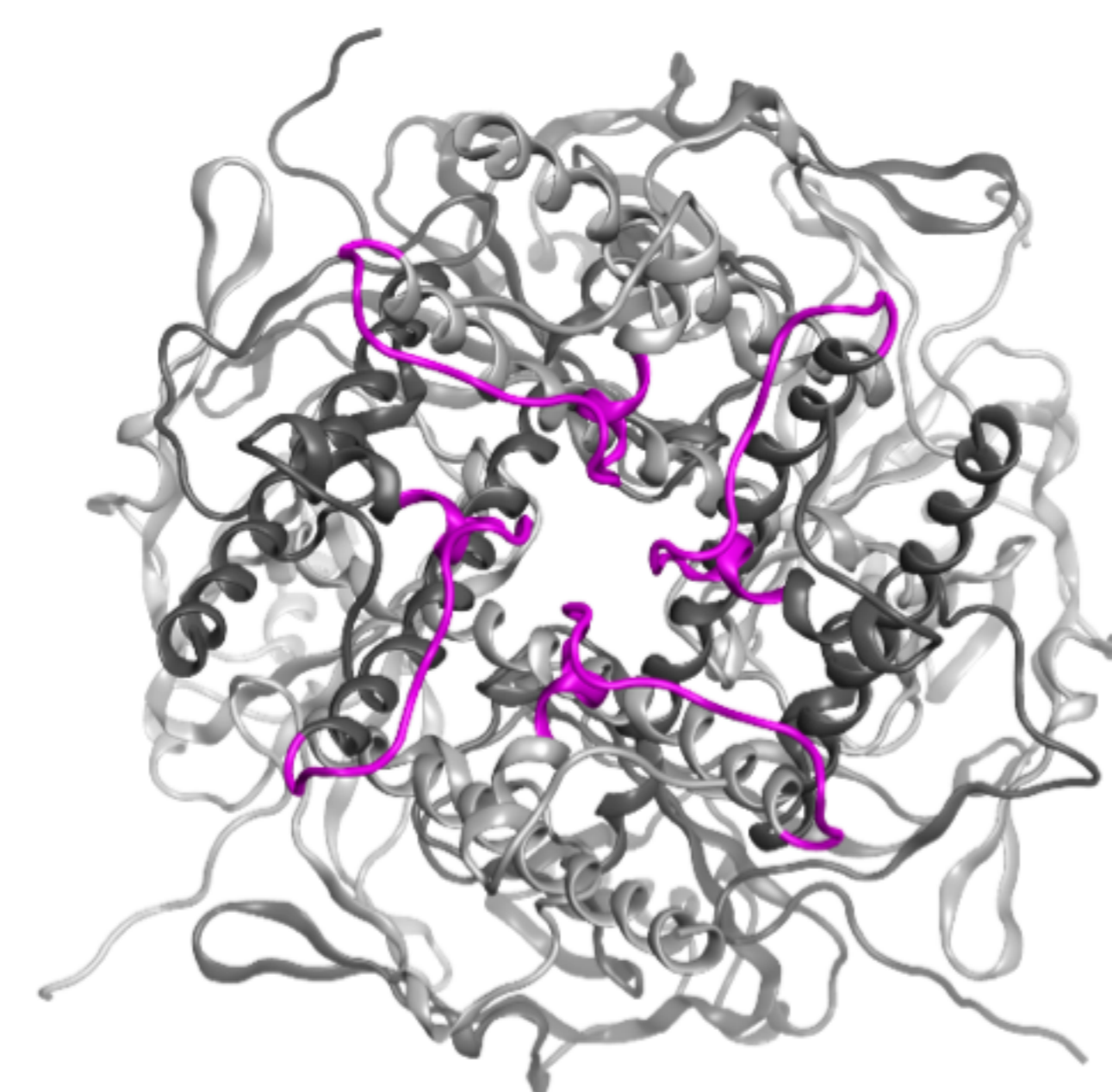

Supplement: Supplementary file 3 [file cir-148-459-s003.pdf]
